# Systemic Ghrelin Administration Alters Serum Biomarkers of Angiogenesis in Diet-Induced Obese Mice

**DOI:** 10.1155/2013/249565

**Published:** 2013-02-28

**Authors:** M. Khazaei, Z. Tahergorabi

**Affiliations:** Department of Physiology, Isfahan University of Medical Sciences, Isfahan 81743638, Iran

## Abstract

*Introduction*. Ghrelin is a gastrointestinal endocrine peptide that was initially identified as the endogenous ligand of growth hormone secretagogue receptor; however, recently, the cardiovascular effect of this peptide has been indicated. In this study, we investigated the effect of ghrelin administration on serum biomarkers of angiogenesis including leptin, nitric oxide (NO), vascular endothelial growth factor (VEGF), and its soluble receptor (VEGF receptor 1 or sFlt-1) in control- and diet-induced obese mice. *Methods*. Male C57BL/6 mice were randomly divided into four groups, normal diet (ND) or control, ND + ghrelin, high-fat-diet (HFD) or obese and HFD + ghrelin (*n* = 6/group). Obese and control groups received either HFD or ND for 15 weeks. Then, the ghrelin was injected subcutaneously 100 *µ*g/kg twice daily for 10 days. At the end of experiment, blood samples were collected for blood glucose, serum insulin, VEGF, sFlt-1, NO, and leptin measurements. *Results*. The obese animals had higher serum NO and leptin concentrations without changes in serum VEGF and sFlt-1 levels compared to control. Administration of ghrelin significantly increased serum VEGF and decreased serum leptin and NO concentrations in HFD group. *Conclusion*. Since ghrelin changes serum biomarkers of angiogenesis, it seems that it gets involved during states with abnormal angiogenesis.

## 1. Introduction

Prolonged imbalance of caloric intake and energy expenditure leads to complex metabolic disorder of obesity. It is associated with most common and chronic human diseases including type 2 diabetes, heart diseases, hypertension, and cancer [1].

Angiogenesis, the formation of new blood vessels from preexisting ones, is tightly linked with adipogenesis [2] and is considered as an essential component in development and expansion of adipose tissue [3]. Since expansion of adipose tissue (increasing cell size and number) creates adipose tissue hypoxia, it can lead to stabilization of the transcription factor hypoxia inducible factor1*α* (HIF-1*α*) [4, 5] that induces an angiogenic response [6].

Ghrelin is a gastrointestinal endocrine peptide and is identified as an endogenous ligand for the growth hormone secretagogue receptor type 1a (GHS-R Ia) [7]; however, it also regulates food intake and is associated with obesity [8]. Ghrelin and its receptors are expressed in endothelial cells and stimulate endothelial cell proliferation, migration, and angiogenesis [9]. Recently, the impact of ghrelin on cardiovascular system has been reported [10] including a decrease of peripheral vascular resistance in consequence an increase in cardiac index and stroke volume [11], improvement of ventricular remodeling [12], protection of myocytes from apoptosis [13], decrease of cardiac injury induced by ischemia/reperfusion (I/R) injury [14], and reduction of the infarct size (L). It also improves endothelial dysfunction, reduces vasoconstrictor effect of endothelin-1, and decreases blood pressure [10].

Plasma ghrelin level is associated with body mass index (BMI). It is indicated that obese patients have reduced plasma ghrelin levels [8]. The main objective of this study was to investigate the effect of ghrelin administration on serum biomarkers of angiogenesis including leptin, nitric oxide (NO), vascular endothelial growth factor (VEGF), and its soluble receptor (VEGF receptor 1 or sFlt-1) in control and obese mice.

## 2. Materials and Methods

### 2.1. Animals

Male C57BL/6 mice (5 weeks old, *n* = 24) were purchased from Pasteur Institute (Tehran, Iran), and three or four animals were housed together in one cage in controlled environment under a light-dark cycle (lights on at 19:00 and off at 07:00). The experimental procedures followed the Guiding Principles for the Care and Use of animals and were approved by the Isfahan University of Medical sciences. All mice were randomly divided into four groups: normal diet (ND) or control, ND + ghrelin, high-fat-diet (HFD) or obese and HFD + ghrelin (*n* = 6/group). 

### 2.2. Diets and Ghrelin Administration

Mice were rendered obese by the HFD (Bio-Serv Research Diets, NJ, USA; Cat #F3282) contained with 59% from fat, 14% from protein, and 27% from carbohydrate (of total calories) starting at 5 weeks of age for 15 weeks. The ND mice were fed a standard diet (Pasteur Institute, Iran). All groups were allowed to eat food freely and had free access to water. Body weights were measured weekly. After 15 weeks, the ghrelin (Tocris Co., Bristol, UK) was administered subcutaneously 100 *μ*g/kg twice daily for 10 days [15, 16].

### 2.3. Serum Measurements

Blood glucose was measured by glucometer (ACON Lab Inc San Diego, CA, USA) ELISA kits were used for determination of mice serum insulin (Mercodia, Uppsala, Sweden), VEGF and sFlt-1 (R&D systems, Minneapolis, USA), leptin (Invitrogen, Camarillo, CA 93012) and nitrite, the main metabolite of NO (Promega Corp, USA) concentrations. 

### 2.4. Statistical Analysis

All values are expressed as mean ± SEM. The statistical software SPSS version 16 was used for data analysis. One-Way ANOVA was used to compare data between groups using LSD post-hoc test. *P* < 0.05 was considered statistically significant. 

## 3. Results

### 3.1. Effect of Ghrelin on Body Weight

**Figure 1:**
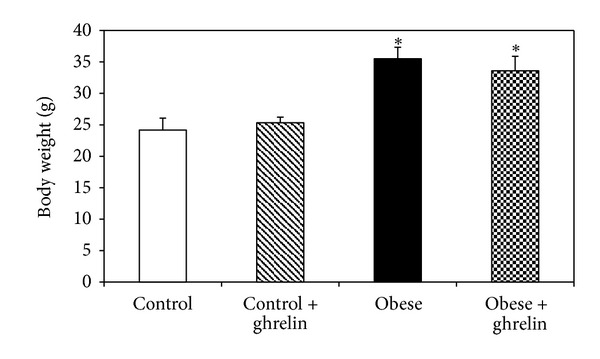
Body weight of the animals at the end of experiment. *P < 0.05 compared to control groups.

Figure 1 illustrates that administration of ghrelin for 10 days did not significantly change body weight in obese and control mice (*P* > 0.05).

### 3.2. Effect of Ghrelin on Blood Glucose and Serum Insulin Levels

**Figure 2:**
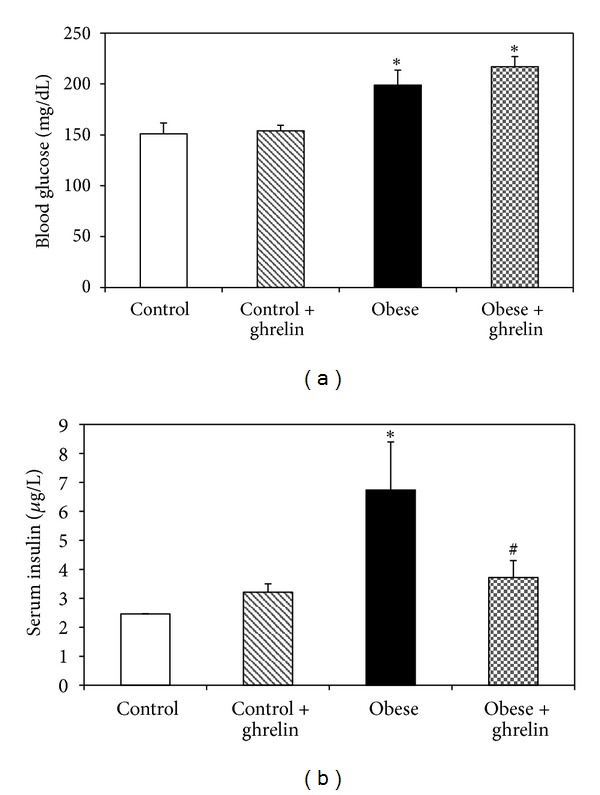
Blood glucose (a) and serum insulin (b) concentrations in experimental groups. *P < 0.05 compared to control. ^#^ *P* < 0.05 compared to obese group.

As shown in Figure 2, there was a significant difference in blood glucose level between obese and control groups (*P* < 0.05). Administration of ghrelin did not significantly change blood glucose in obese and control mice (*P* > 0.05).

Serum insulin concentration in obese mice was significantly higher than that of control (*P* < 0.05). Ghrelin administration did not alter serum insulin concentration in control groups (*P* > 0.05), while significantly reduced it in obese group (*P* > 0.05) (Figure 2).

### 3.3. Effect of Ghrelin on Serum Biomarkers of Angiogenesis

**Figure 3:**
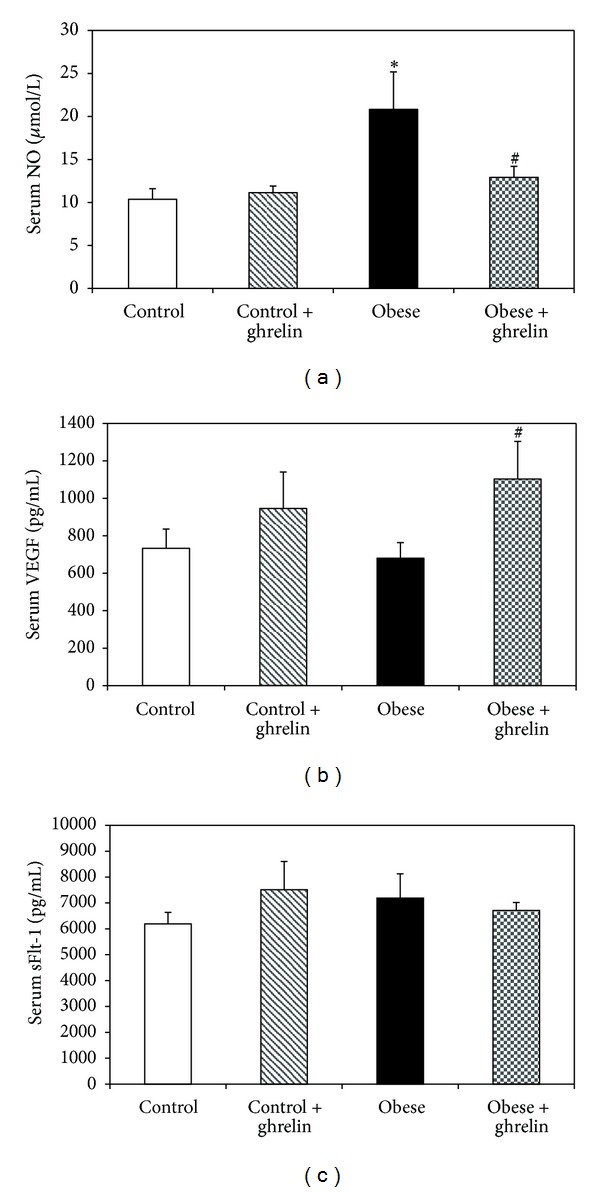
Effect of ghrelin on serum NO (a), VEGF (b), and sFlt-1 (c) concentrations. *P < 0.05 compared to control. ^#^ *P* < 0.05 compared to obese group.

The results indicated no significant differences in serum VEGF and sFlt-1 between obese and control animals (*P* < 0.05); however, serum NO concentration in obese mice was higher than that of control (*P* < 0.05). Ghrelin administration increased serum VEGF and reduced serum NO level in obese mice and had no effect on sFlt-1 concentration (Figure 3). 

### 3.4. Serum Leptin Measurement

**Figure 4:**
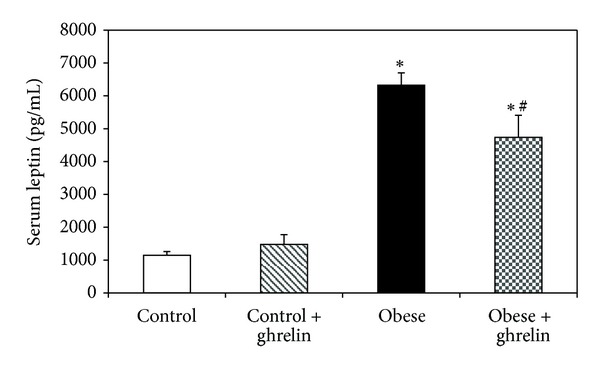
Effect of ghrelin on serum leptin level. *P* < 0.05 compared to control. *P < 0.05 compared to control. ^#^ *P* < 0.05 compared to obese group.

Serum leptin level in obese mice was higher than that of control (*P* < 0.05), and ghrelin significantly reduced it in obese group (*P* < 0.05) (Figure 4). 

## 4. Discussion

The main finding of this study is that the obese mice had higher serum insulin, NO, and leptin concentrations compared to control without changes in serum VEGF and sFlt-1 levels. Ghrelin administration reduced serum NO, and leptin and increased serum VEGF concentrations in obese mice.

Higher blood glucose and insulin levels in HFD group indicate the insulin resistance in these animals. We demonstrated that although ghrelin treatment could not alter blood glucose level, it reduced serum insulin concentration in obese mice. Our data was in line with other studies [17, 18]. Ghrelin may also act on cellular glucose uptake [10] and may involve in control of glucose metabolism and insulin sensitivity [19]. Ghrelin stimulates insulin release; however, leptin inhibits insulin [20]. Perhaps, only ten days ghrelin treatment was the reason for unchanging of blood glucose level in the present study.

Modulation of vascular tissue and angiogenesis in adipose tissue is a strategy to affect obesity. Adipose tissue endothelial cells produce several angiogenic factors including leptin, NO, VEGF, FGF, HGF, and other growth factors [21]. NO is an endothelium-derived relaxing factor which has antiatherosclerotic effects through different mechanisms. However, it is a known angiogenic factor [22]. It is suggested that at the initial stage of obesity, a compensatory increase in NO production occurs due to upregulation of NO synthase [23]. On the other hand, adipogenesis increases upregulation of iNOS which increases NO synthesis due to chronic low-grade inflammation during obesity [2]. These data are in line with the results of the present study that we showed higher serum NO concentration in obese mice.

Leptin is an adipocyte-derived hormone that not only directly promotes angiogenesis and endothelial cell migration but also upregulates VEGF expression [24]. As we expected, in the present study, the obese animals had higher serum leptin level than that of control. These data was in agreement with the previous studies [25]. We also demonstrated that HFD did not change serum VEGF and sFlt-1 concentrations. Although some studies indicated higher serum VEGF level in obese subjects [26], a recent study showed that HFD did not affect plasma concentration of VEGF [27]. VEGF binds to two tyrosine kinase receptors of sFlt-1 and VEGFR2. sFlt-1 leads to anti- or proangiogenic signaling and inhibits angiogenic signaling through sequestration of VEGF ligands [28, 29]. In the present study, HFD did not change serum concentration of sFlt-1. 

In our study, ghrelin administration reduced serum NO and leptin and increased serum VEGF concentrations in obese mice. Ghrelin is a gastrointestinal endocrine peptide which has several impacts on cardiovascular system [10]. Ghrelin and leptin circulate in the blood and have a role in regulation of body weight and energy homeostasis [13]. Study in human showed that plasma ghrelin inversely correlated to degree of obesity [30] and in this study, ghrelin reduced serum leptin level in obese mice. Thus, it seems that ghrelin has a protective mechanism including leptin resistance in setting obesity. Further studies need to clarify this. An *in vitro* studies indicated that ghrelin activates NO-dependent vasorelaxation in patients with metabolic syndrome [31]. Furthermore, there is a reciprocal regulation between VEGF and NO during angiogenesis process [32]. Thus, we expected that in the present study, ghrelin administration increased serum NO concentration. One explanation for this discrepancy is that ghrelin and leptin have mutually antagonistic effects on inflammatory cytokine expression in obesity [33] and reduced leptin after ghrelin administration may involve in reduction of serum NO level.

Recently, Yuan M.J. showed that in a rat model of myocardial infarction, chronic ghrelin treatment increased VEGF expression in peri-infarct zone and they suggested that ghrelin may induce angiogenesis after MI [34]. We also found that ghrelin altered serum biomarkers of angiogenesis and it seems that it may mediate angiogenesis through different mechanisms. Taken together, our results suggested that ghrelin administration changes the serum biomarkers of angiogenesis and can be involved during states with abnormal angiogenesis.
